# Antidepressant-like activity of magnesium in the olfactory bulbectomy model is associated with the AMPA/BDNF pathway

**DOI:** 10.1007/s00213-014-3671-6

**Published:** 2014-07-16

**Authors:** Bartlomiej Pochwat, Magdalena Sowa-Kucma, Katarzyna Kotarska, Paulina Misztak, Gabriel Nowak, Bernadeta Szewczyk

**Affiliations:** 1 Department of Neurobiology, Institute of Pharmacology, Polish Academy of Sciences, Smetna 12, 31-343 Krakow, Poland; 2 Centre of Applied Biotechnology and Basic Sciences, University of Rzeszów, Rejtana 16c, 35-959 Rzeszów, Poland; 3 Department of Genetics and Evolution, Institute of Zoology, Jagiellonian University, Gronostajowa 9, 30-387 Krakow, Poland; 4 Department of Pharmacobiology, Faculty of Pharmacy, Jagiellonian University Medical College, Medyczna 9, 30-688 Krakow, Poland

**Keywords:** Olfactory bulbectomy, Magnesium, NMDA, AMPA, BDNF, Glutamatergic system, Depression

## Abstract

**Rationale:**

Numerous studies suggest agents that act on glutamatergic transmission as potential antidepressants. Preclinical and clinical evidence suggests that magnesium, an *N*-methyl-d-aspartate receptor blocker, may be useful in the treatment of depression.

**Objective:**

The aim of this study was to investigate the effects of magnesium on behavior; protein levels of GluN2A, GluN2B [*N*-methyl-d-aspartate receptor (NMDAR) subunits], GluA1 [α-amino-3-hydroxy-5 methyl-4-isoxazolepropionic acid (AMPA) subunit], phospho-Ser-831-GluA1 (P-S831), phospho-Ser-845-GluA1 (P-S845), and brain-derived neurotrophic factor (BDNF); and messenger RNA (mRNA) levels of GluN2A and GluN2B in different brain areas in the olfactory bulbectomy (OB) model of depression in rats.

**Methods:**

Magnesium was administered once daily for 14 days at three doses (10, 15, and 20 mg/kg, intraperitoneal) to sham and OB rats. Following treatment, open field and passive avoidance tests were performed in the sham and OB rats. After 24 h, the hippocampus, the prefrontal cortex (PFC), and the amygdala of rats treated with the most active dose (15 mg/kg) were harvested, and the protein and mRNA levels were determined.

**Results:**

Chronic administration of magnesium (15 and 20 mg/kg) reduced the number of trials required to learn passive avoidance and reduced the OB-induced hyperactivity. OB increased the P-S845 level in the hippocampus, which was reduced by magnesium treatment. Magnesium significantly increased the levels of BDNF, GluN2B, P-S831, and P-S845 protein (and mRNA) primarily in the PFC and the hippocampus in OB rats.

**Conclusion:**

For the first time, the present results demonstrate the antidepressant-like activity of magnesium in the OB animal model of depression and indicate the potential involvement of the AMPA/BDNF pathway in this activity.

## Introduction

Numerous recent studies have indicated agents that act on glutamatergic transmission function as potential alternative approaches in the pharmacological treatment of depression (Li et al. 2010; Autry et al. 2011; Lima-Ojeda et al. 2013). The most promising results have arisen from trials with *N*-methyl-d-aspartate receptor (NMDAR) antagonists. The antidepressant activity of ketamine, a nonselective antagonist of NMDARs, has been known for many years (Berman et al. 2000). The clinical effects of ketamine have been observed within hours and occur in patients resistant to typical antidepressant drugs and in patients with resistance in bipolar disorder (Zarate et al. 2006, 2012). The activation of glutamatergic transmission achieved by ketamine leads to several changes, such as the potentiation of α-amino-3-hydroxy-5 methyl-4-isoxazolepropionic acid receptor (AMPARs), the activation of the mammalian target of rapamycin (mTOR), and an intensified release of brain-derived neurotrophic factor (BDNF) (Li et al. 2010). The potentiation of AMPARs depends on numerous modifications that change the receptor’s functional state; the most well-known modifications include the phosphorylation of S-831of Ca2+/calmodulin (CaMKII), protein kinase C (PKC), and S-845 protein kinase A (PKA) (Lee et al. 2010; Lee and Kirkwood. 2011).

The functional state of NMDARs is also regulated by natural factors, such as magnesium and zinc (Szewczyk et al. 2012). Blockade of the NMDAR’s ion channel is the most established mechanism involved in magnesium’s action in the central nervous system (CNS) (Paoletti et al. 1995). In clinical studies, many patients with mood disorders have altered plasma/serum magnesium concentrations (Serefko et al. 2013). A lower level of magnesium has been identified in patients with long-lasting and unipolar depression (Kirov et al. 1994). In cases of acute depression, such effects have not been observed (Linder et al. 1989; Hashizume and Mori 1990).

The importance of magnesium for the normal functioning of the CNS has also been corroborated in preclinical studies that have revealed a deficiency of magnesium induces depressive-like behavior and anxiety that are reversed by antidepressant treatment (Singewald et al. 2004). Preclinical studies have also indicated that magnesium is an antidepressant agent that acts through the NMDA pathway. It has been demonstrated that the antidepressant-like activity of magnesium in the forced swim test (FST) was antagonized by agonists of NMDARs, such as d-serine and NMDA. Furthermore, the administration of ineffective doses of magnesium in combination with ineffective doses of NMDAR antagonists (e.g., CGP 37849, d-cycloserine, L-701,324, or MK-801) has been shown to cause a significant reduction in the immobility time in the FST (Decollogne et al. 1997; Poleszak et al. 2007, 2008). The hypothesis that magnesium is a potential agent in the treatment of depression is also enhanced by our previous studies that evaluated magnesium efficacy in the chronic mild stress (CMS) model of depression. This study examined the activity of magnesium in the sucrose intake test, which measures anhedonia as the core symptom of depression (Pochwat et al. 2014).

Our present study examined the antidepressant potential of magnesium (10, 15, and 20 mg/kg) in the bilateral olfactory bulbectomy model (OB), which is a widely accepted animal model of agitated depression (Kelly et al. 1997); importantly, agitated depression is a major risk factor for suicide (Rihmer 2007). Behavioral changes and the affinity of magnesium to reverse abnormalities caused by the removal of the olfactory bulbs were determined in the open field and passive avoidance tests. For the biochemical assays, we selected the dose of 15 mg/kg, which is the dose active in both tests as well as in the CMS paradigm (Pochwat et al. 2014). Autoradiography data from animal studies show a reduced level of NMDARs in OB rats (Robichaud et al. 2001), and the other study suggested that OB reduced the potency of glycine to inhibit [3H] 5,7-DCKA binding (Nowak 1996). As a result of this and other studies that indicated OB influence on ionotropic glutamate receptors (Song and Leonard 2005), we directed our efforts to study the changes in the protein levels of GluN2A, GluN2B subunits of NMDARs, and GluA1 subunit of AMPARs. Additional evidence to support this approach was obtained from human postmortem data that demonstrated decreased hippocampal and cortical levels of the GluN2B subunit of NMDARs in patients with MDD (Feyissa et al. 2009; Sowa-Kucma et al. 2013). In view of the crucial role of the GluA1 subunit in the control level of BDNF (Li et al. 2010) and the antidepressant-like activity of NMDAR antagonists, the levels of phospho-Ser-831-GluA1 (P-S831) and phospho-Ser-845-GluA1 (P-S845) of the GluA1 subunit and BDNF were also determined. We performed all assays in the PFC, the hippocampus, and the amygdala, which are critical structures for the development of human depression (Nestler et al. 2002) and depressive-like symptoms in OB rats (Song and Leonard 2005). For comparison with protein expression, we evaluated the expression levels of the GluN2A and GluN2B mRNA subunits in the PFC and the hippocampus.

## Material and methods

### Animals and housing

All procedures were conducted according to the National Institute of Health Animal Care and Use Committee guidelines and were approved by the Ethical Committee of the Institute of Pharmacology, Krakow. The experiments were performed on male Sprague-Dawley rats (220–250 g). The animals were maintained on a normal day-night cycle (light phase 7:00–19:00) and temperature (19–21 °C) with free access to food and water. Each experimental group consisted of six to eight animals. All animals were first subjected to the open field test and to the passive avoidance test 2 days later. The experiments were performed during the light period (9:00–14:00 h).

### Olfactory bulbectomy—surgical procedure

One week after their arrival to the laboratory, a bilateral olfactory bulbectomy was performed on the rats under ketamine (100 mg/kg)/xylazine (10 mg/kg) anesthesia. Metoxicam (0.05 mg/kg, subcutaneous) was administered as an analgesic and anti-inflammatory drug prior to the operation and 2 days after the surgery. Following the exposure of the skull, burr holes were drilled (7 mm anterior to the bregma). The olfactory bulbs were removed by suction, and the burr holes were filled with a hemostatic sponge (Ferrosan, Poland). The skin was then closed. The sham-operated animals were treated similarly, but the bulbs were left intact. The animals were allowed to recover for 14 days following surgery. During this time, they were handled daily by the experimenter to prevent the development of aggressive behavior.

**Fig. 1:**
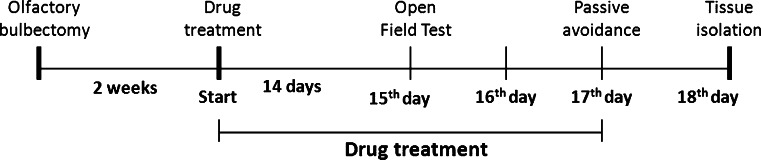
Schedule of the experimental procedure

Amitriptyline (10 mg/kg) and magnesium hydroaspartate (10, 15, and 20 mg/kg, calculated as magnesium ions) were chronically administered once daily for 14 days between 8:00 and 10:00 a.m. via an intraperitoneal (i.p.) injection. Twenty-four hours after the last dose, the open field test was performed. Drug treatment was continued to the next day. The passive avoidance test was conducted 2 days after the open field test and 24 h after drug administration (Fig. 1). The control animals received a vehicle solution (0.9 % sodium chloride). The drugs were injected at a constant volume of 2 ml/kg.

### Open field test

The open field test was performed using an “open field” apparatus (an arena 90 cm in diameter, divided into 10 cm squares by faint yellow lines). The arena was surrounded by a 75-cm high aluminum sheet. All measurements were performed in a darkened room. Illumination was provided by a 60-W bulb which was positioned 90 cm above the floor. Each rat was placed individually in the center of the open field and allowed to freely explore it for 3 min. The behaviors of interest included episodes of rearing, defined as raising the forepaws from the floor, and ambulation, defined as the number of sector lines crossed (once a line had been crossed with all four paws).

### Passive avoidance test

The passive avoidance test was performed using a Plexiglas box (50 cm × 50 cm × 50 cm) with a grid floor. The grid floor consisted of parallel steel rods set 1.2 cm apart. A wooden platform (12 cm × 12 cm × 4 cm) was placed in the center of the grid floor where the rats were placed individually. Once the rat left the platform with all four paws, it received an electric shock 0.75 mA/1 s). The animals were then immediately removed from the experimental cage and transferred to the home cage. After 30 s, the next trial on the same rats was initiated. The training was stopped if the rats learned to not leave the platform before the passage of 1 min or if 15 trials occurred.

### Western blot analysis

Twenty-four hours after the last dose of magnesium, the animals were killed and their brains were collected. The hippocampus, the amygdala, and the prefrontal cortex were rapidly dissected. The tissues were frozen on dry ice and stored at −80 °C. In the next step, they were homogenized in ice in a 2 % solution of sodium dodecyl sulfate (SDS). The homogenates were subsequently denatured at 95 °C for 10 min and centrifuged for 5 min at 10,000 rpm at 4 °C. After centrifugation, the supernatant was collected and the protein content was determined. For this assay, bicinchonic acid was used (Pierce). Next, the samples were fractionated by 10 % SDS-polyacrylamide gel electrophoresis. In a further step, the proteins were transferred to a nitrocellulose membrane (Invitrogen, Paisley, UK). To block nonspecific binding, a 1 % blocking solution was used (BM Chemiluminescence Western Blotting Kit (Mouse/Rabbit), Roche). After blocking, the membranes were incubated overnight at 4 °C with the respective antibodies. The following antibodies were used: polyclonal anti-NMDAR2A antibody (Abcam), diluted 1:1,000; polyclonal anti-NMDAR2B antibody (Abcam), diluted 1:1,000; polyclonal AMPAR GluA1 antibody (Abcam), monoclonal anti-P-S831, and monoclonal anti-P-S845 (Abcam), all diluted 1:1,000; and polyclonal BDNF (Santa Cruz Biotechnology), 1:500 diluted. All antibodies were dissolved in 0.5 % blocking reagent (Roche). The next day, the membranes were washed three times for 10 min in Tris-buffered saline with Tween (TBS-T) and incubated for 30 min with anti-mouse IgG-peroxidase-conjugated anti-rabbit-IgG-peroxidase-conjugated antibodies (diluted 1:7,000). This set of secondary antibodies was also a component of the BM Chemiluminescence Western Blotting Kit (Mouse/Rabbit) (Roche). After incubation, the membranes were washed three times for 10 min with TBS-T. In the last step, the blots were incubated with a detection reagent (Roche). The signal from the tested proteins was visualized and measured using the Fuji-Las 1000 system and Fuji Image Gauge v.4.0 software (Fig. 10). To confirm the transfer and loading, β-actin was indicated on each blot using a primary monoclonal antibody (Millipore; 1:8,000; Fig. 10). The procedures were the same for the other proteins. The final results are provided as the ratio of the optical density of specific proteins to the optical density of β-actin.

### RNA isolation and real-time RT-PCR

The tissue samples were homogenized in TissueLyser (Qiagen, Germany) and immediately subjected to RNA isolation with TRIzol reagent (Invitrogen). The integrity of the obtained RNA was confirmed by gel electrophoresis. The RNA purity and concentration were assessed with a Nanodrop spectrophotometer (Thermo Scientific, USA). One microgram of total RNA from each sample was digested with DNase I (Sigma-Aldrich, Germany) and reverse-transcribed into complementary DNA (cDNA) using the High Capacity cDNA Reverse Transcription Kit with random hexamers (Applied Biosystems, USA). Real-time polymerase chain reactions (PCRs) were conducted on a CFX96 Real-Time System (Bio-Rad, USA) and C1000 Touch Thermal Cycler (Bio-Rad) using 96-well optical plates (Bio-Rad). The 18-μl PCR reaction mixtures included the following: 1.8 μl of cDNA sample (diluted 1:10 in RNase-free water), Power SYBR Green Master Mix (Applied Biosystems), and primers in concentrations of 200 nM each. The primer sequences are shown in Table 1. The Basic Local Alignment Search Tool (BLAST) from the National Center for Biotechnology Information (NCBI) was used to preclude the homology of the primers used for any other sequences in the database. The PCR reactions were incubated at 95 °C for 10 min followed by 40 cycles at 95 °C for 15 s and 60 °C for 1 min. Then, a melt curve was drawn for each primer pair to ensure that there was no primer–dimer formation. Glyceraldehyde-3-phosphate dehydrogenase (*Gapdh*) was used as the endogenous reference gene. All reactions were performed in triplicate, and the average threshold cycle (*C*_T_) values were calculated. For each sample, the *C*_T_ value of the endogenous reference gene was subtracted from the *C*_T_ values of the target genes to obtain the Δ*C*_T_ values and to normalize the PCRs for the amount of cDNA added to the subsequent reactions. For graphical presentation and statistical analysis, relative mRNA level indexes of GluN2A and GluN2B genes were generated with the 2^−ΔCT^ formula (Livak and Schmittgen 2001).

**Table 1:** Real-time PCR primers used in this study (5' to 3')

Primers	Forward primer sequence	Reverse primer sequence
GluN2A	ACATTGCAGAAGCTGCCTTT	TTCTGTGACCAGTCCTGCTG
GluN2B	GTGAGAGCTCCTTTGCCAAC	TGAAGCAAGCACTGGTCATC
GAPDH	AGACAGCCGCATCTTCTTGT	CTTGCCGTGGGTAGAGTCAT

### Statistical analysis

All of the results are shown as the means ± SEM; the data were analyzed by two-way ANOVA followed by the Newman–Keuls test (behavioral studies) or the Bonferroni multiple comparison test (biochemical studies). *p* < 0.05 was considered statistically significant.

## Results

### Effects of OB and the administration of magnesium on passive avoidance acquisition and number of ambulations in the open field test

**Fig. 2:**
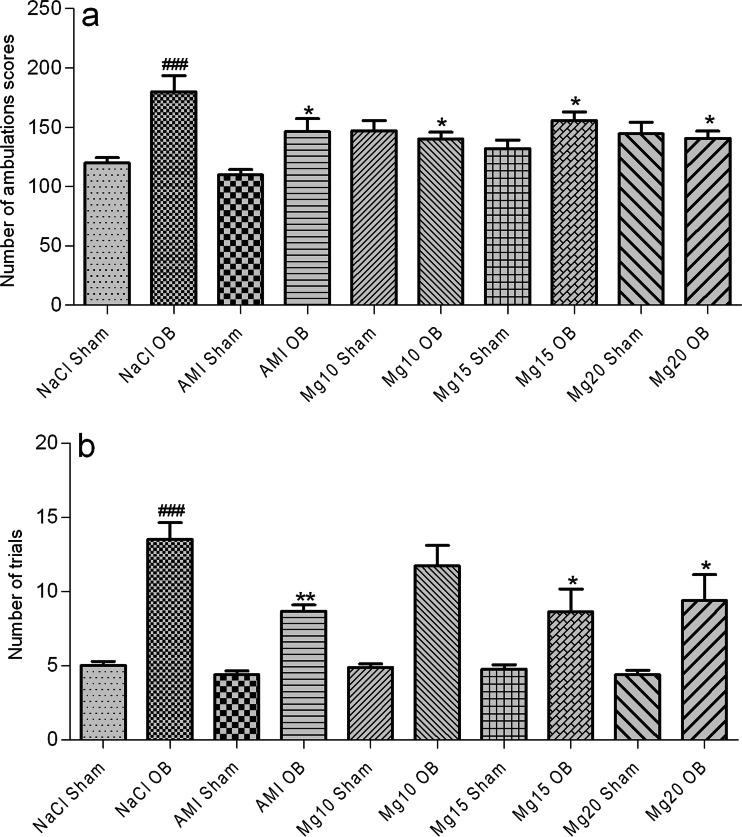
The effect of amitriptyline (10 mg/kg) and magnesium (10, 15, and 20 mg/kg, i.p.) treatment on the activity of rats in the open field test (**a**) and the number of trials in the passive avoidance test (b). All compounds were administered for 14 days. On the 15th day of chronic amitriptyline and magnesium treatment, the open field test followed by the passive avoidance test (next day) was performed. The values represent the mean ± SEM, *n*=7-8 and were analyzed by one-way ANOVA followed by Newman–Keuls test. ###*p* < 0.001, relative to NaCl sham; **p < 0.01, *p < 0.05 relative to NaCl OB. Seven to eight animals per group were used

As shown in Fig. 2a, the OB procedure increased the activity of the rats in the open field test, which was expressed as an increase in the number of ambulations (###*p* = 0.0002 NaCl sham vs. NaCl OB). An amitriptyline dose of 10 mg/kg and magnesium doses of 10, 15, and 20 mg/kg reversed this effect in the OB animals (*p = 0.03 AMI OB vs. NaCl OB; *p = 0.01 Mg10 OB vs. NaCl OB; *p = 0.04 Mg15 OB vs. NaCl OB; *p = 0.01 Mg20 OB vs. NaCl OB). Chronic treatment with amitriptyline or magnesium had no effect in the sham group. Two-way ANOVA demonstrated a significant effect of OB [*F*(1, 64) = 17.15, *p* = 0.0001], no significant effect of treatment [*F*(4, 64) = 1.64, *p* = 0.176], and a significant interaction [*F*(4, 64) = 5.73, *p* = 0.0005].

As shown in Fig. 2b, the removal of olfactory bulbs impaired passive avoidance acquisition (###*p*=0.0001 NaCl sham vs. NaCl OB). Chronic treatment with an amitriptyline dose of 10 mg/kg and magnesium doses of 15 and 20 mg/kg significantly reduced the number of trials required to learn passive avoidance in the OB animals (**p = 0.002 AMI OB vs. NaCl OB; *p = 0.0.01 Mg15 OB vs. NaCl OB; *p=0.01 Mg20 OB vs. NaCl OB). Two-way ANOVA demonstrated significant effects of OB [*F*(1, 65)=85.32, *p*=0.0001] and treatment [*F*(4, 65)=2.89, *p* = 0.029], but no significant interaction [*F*(4, 65)=1.91, *p* = 0.119].

### Effects of OB and administration of magnesium on the GluN2A subunit level

**Fig. 3:**
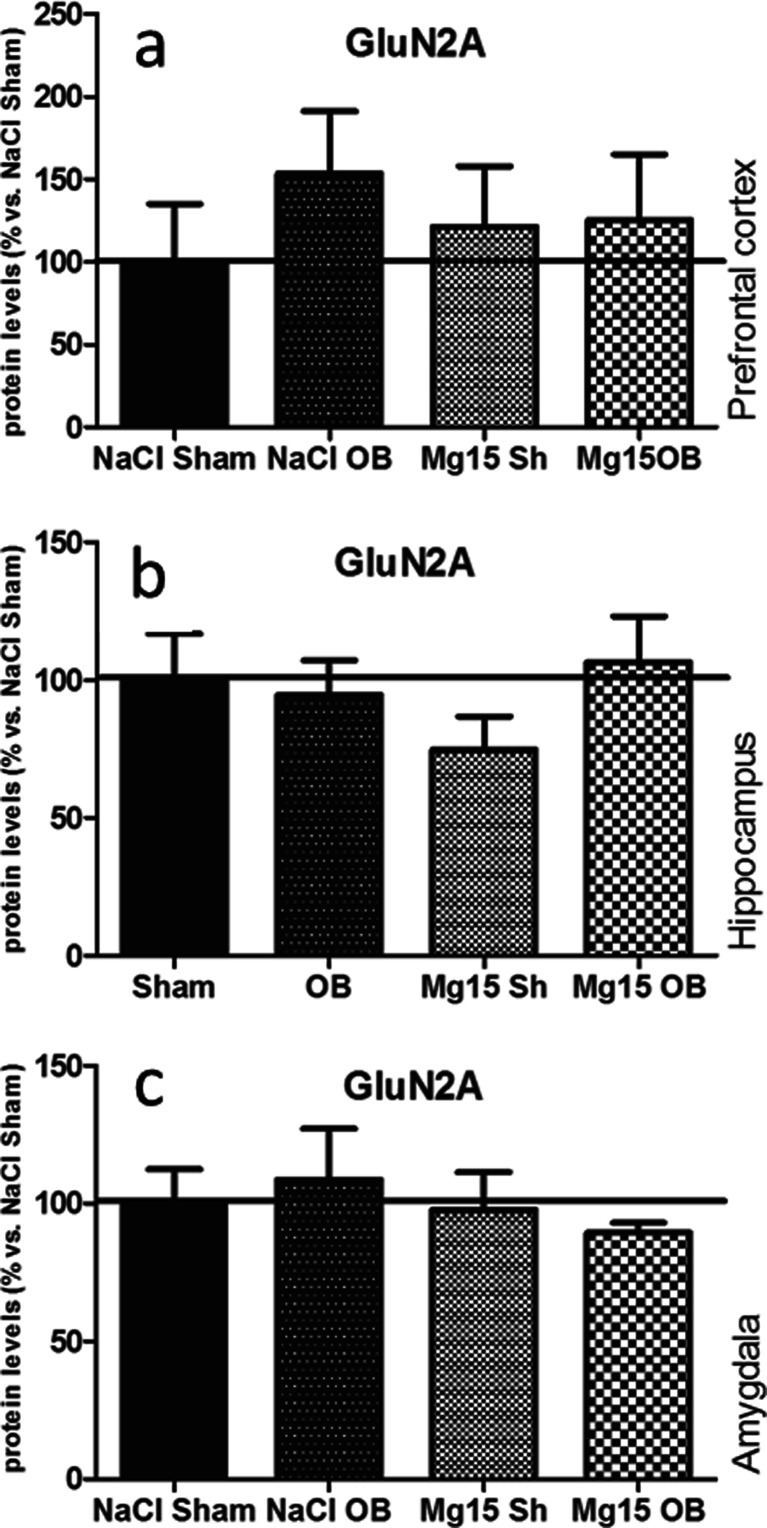
Changes in the protein levels of the GluN2A subunit of the NMDA receptor induced by OB and magnesium treatment. The levels of the GluN2A subunit were measured in the prefrontal cortex (**a**), the hippocampus (**b**), and amygdala (**c**) of sham and OB rats, treated for 2 weeks with a vehicle or magnesium. The values represent the mean ± SEM (*n*=7–8)

As shown in Fig. 3, the OB procedure and magnesium treatment did not induce significant effects in the GluN2A subunit levels in the PFC (a), the hippocampus (b), or the amygdala (c). In the prefrontal cortex, two-way ANOVA indicated that there were no significant effect of OB [*F*(1, 18) = 0.57, *p* = 0.4590] or magnesium [*F*(1, 18) = 0.01, *p* = 0.9302] and no significant interaction [*F*(1, 18) = 0.43, *p* = 0.5220]. In the hippocampus, two-way ANOVA indicated that there were no significant effect of OB [*F*(1, 22) = 0.79, *p* = 0.3837] or magnesium [*F*(1, 22) = 0.2, *p* = 0.6584] and no significant interaction [*F*(1, 22) = 1.56, *p* = 0.2243]. In the amygdala, two-way ANOVA indicated that there were no significant effect of OB [*F*(1, 20 = 0.0, *p* = 0.9868] or magnesium [*F*(1, 20) = 0.58, *p* = 0.4555] and no significant interaction [*F*(1, 20) = 0.38, *p* = 0.5454].

### Effects of OB and administration of magnesium on the GluN2B subunit level

**Fig. 4:**
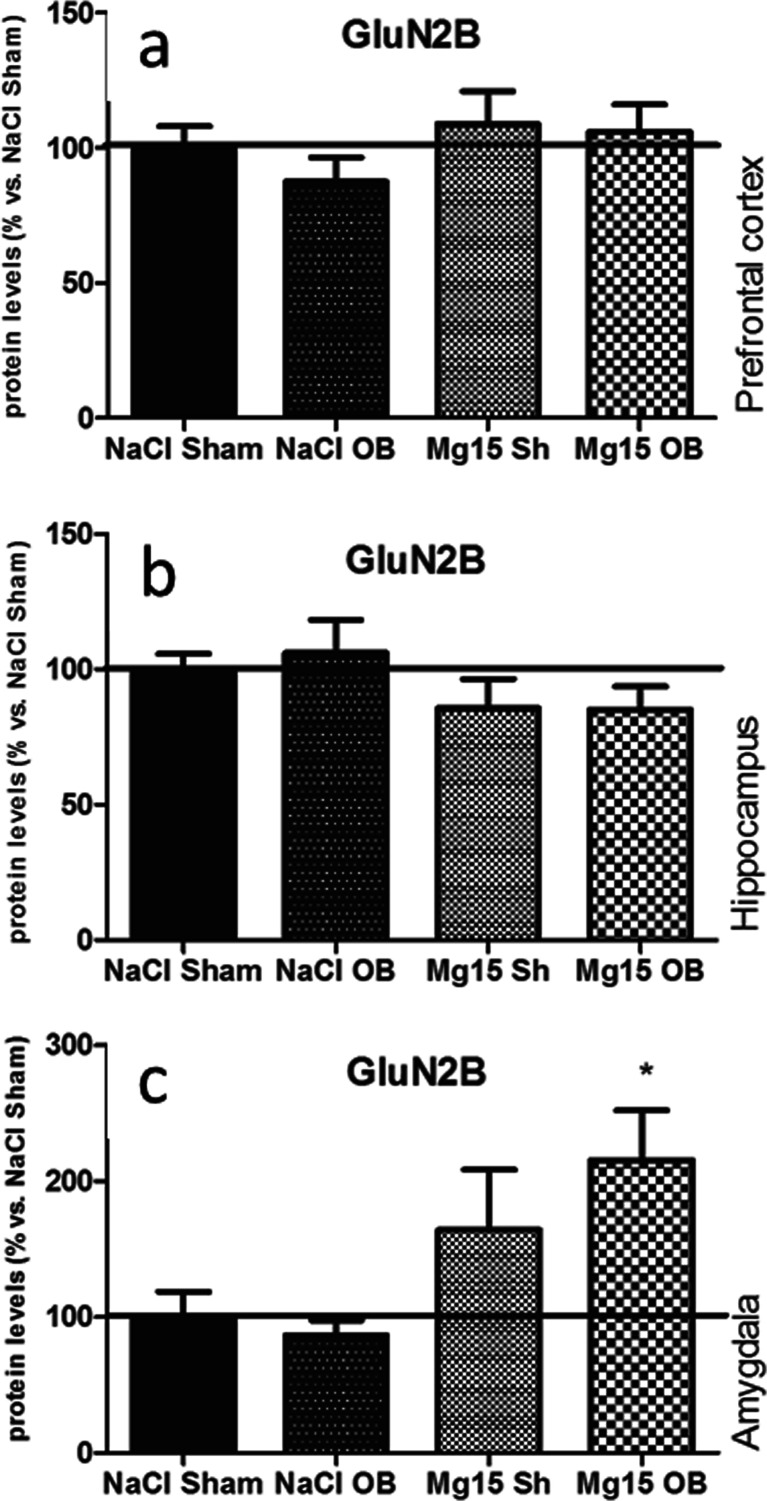
Changes in the protein levels of the GluN2B subunit of the NMDA receptor induced by OB and magnesium treatment. The levels of the GluN2B subunit were measured in the prefrontal cortex (**a**), the hippocampus (**b**), and amygdala (**c**) of sham and OB rats, treated for 2 weeks with a vehicle or magnesium. The values represent the mean ± SEM (*n* = 7–8). *p < 0.05 vs. OB

As shown in Fig. 4, the OB procedure and magnesium treatment did not induce significant effects in the GluN2B subunit levels in the PFC (a), the hippocampus (b), or the amygdala (c) with one exception, that is, when magnesium treatment in the OB animals induced an increased GluN2B subunit level in the amygdala (215 % of NaCl sham, *p* < 0.05 vs. NaCl OB, Fig. 4c). In the PFC, two-way ANOVA indicated that there were no significant effect of OB [*F*(1, 27 = 0.6, *p* = 0.4443] or magnesium [*F*(1, 27) = 1.90, *p* = 0.1791] and no significant interaction [*F*(1, 27) = 0.23, *p* = 0.6382]. In the hippocampus, two-way ANOVA indicated that there were no significant effect of OB [*F*(1, 21) = 0.08, *p* = 0.779] or magnesium [*F*(1, 21) = 3.44, *p* = 0.0776] and no significant interaction [*F*(1, 21) = 0.12, *p* = 0.73]. In the amygdala, two-way ANOVA indicated that there were no significant effect of OB [*F*(1, 21) = 0.39, *p* = 0.5371], a very significant effect of magnesium [*F*(1, 21 = 10.21, *p* = 0.0043], and no significant interaction [*F*(1, 21) = 1.15, *p* = 0.2962].

### Effects of OB and administration of magnesium on the GluA1 subunit level

**Fig. 5:**
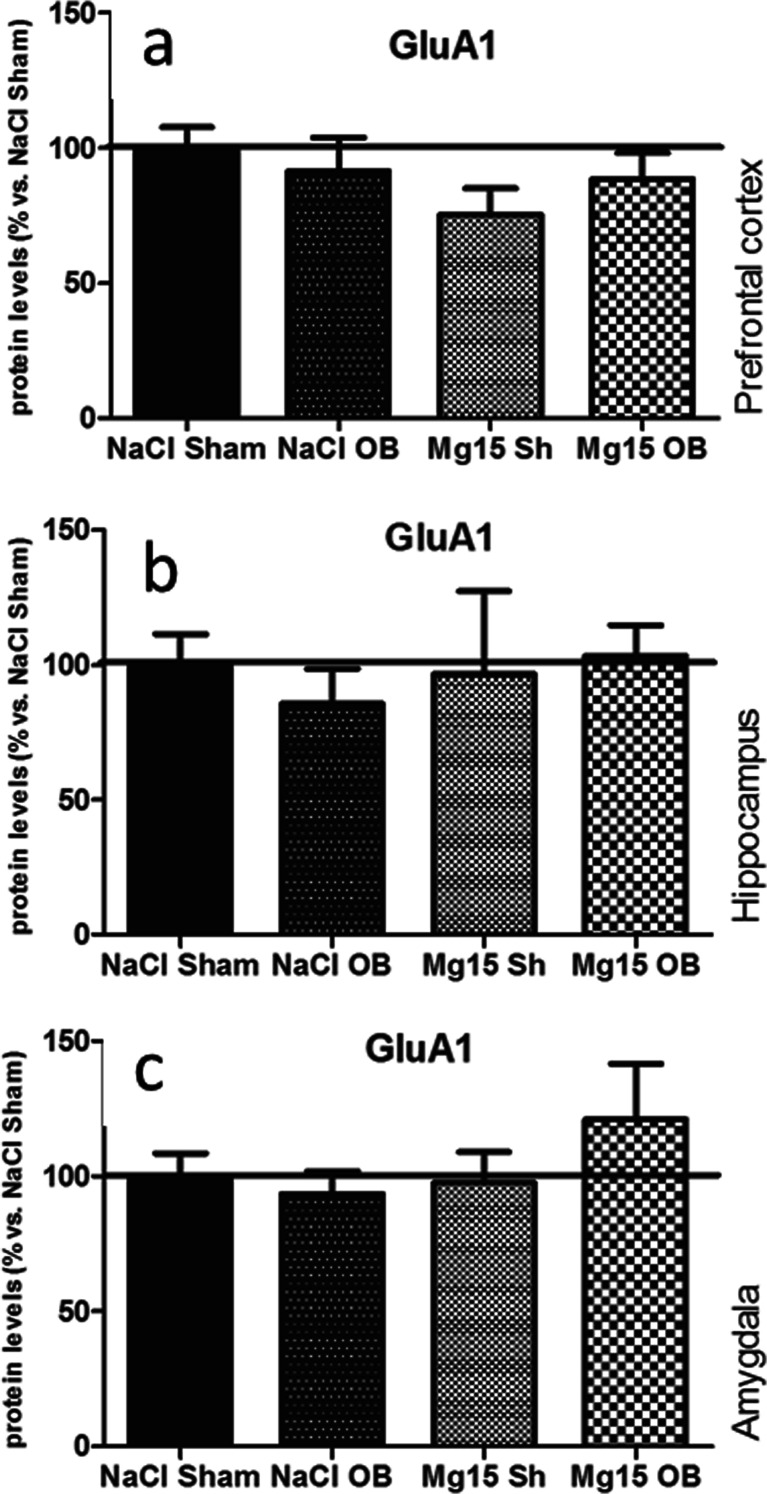
Changes in the protein levels of the GluA1 subunit of the AMPA receptor induced by OB and magnesium treatment. The levels of the GluA1 subunit were measured in the prefrontal cortex (**a**), the hippocampus (**b**), and amygdala (**c**) of sham and OB rats, treated for 2 weeks with a vehicle or magnesium. The values represent the mean ± SEM (*n* = 7–8)

As shown in Fig. 5, the OB procedure and administration of magnesium did not induce significant effects in the GluA1 subunit levels in the PFC (a), the hippocampus (b), or the amygdala (c). In the PFC, two-way ANOVA indicated that there were no significant effect of OB [*F*(1, 26) = 0.05, *p* = 0.8192] or magnesium [*F*(1, 26) = 1.81, *p* = 0.1841] and no significant interaction [*F*(1, 26) = 1.15, *p* = 0.2929]. In the hippocampus, two-way ANOVA indicated that there were no significant effect of OB [*F*(1, 21 = 0.05, *p* = 0.82] or magnesium [*F*(1, 21) = 0.19, *p* = 0.6668] and no significant interaction [*F*(1, 21) = 0.41, *p* = 0.5312]. In the amygdala, two-way ANOVA indicated that there were no significant effect of OB [*F*(1, 27 = 0.45, *p* = 0.5090] or magnesium [*F*(1, 27) = 0.99, *p* = 0.3283] and no significant interaction [*F*(1, 27) = 1.42, *p* = 0.2432].

### Effects of OB and administration of magnesium on the P-S831 of the GluA1 subunit level

**Fig. 6:**
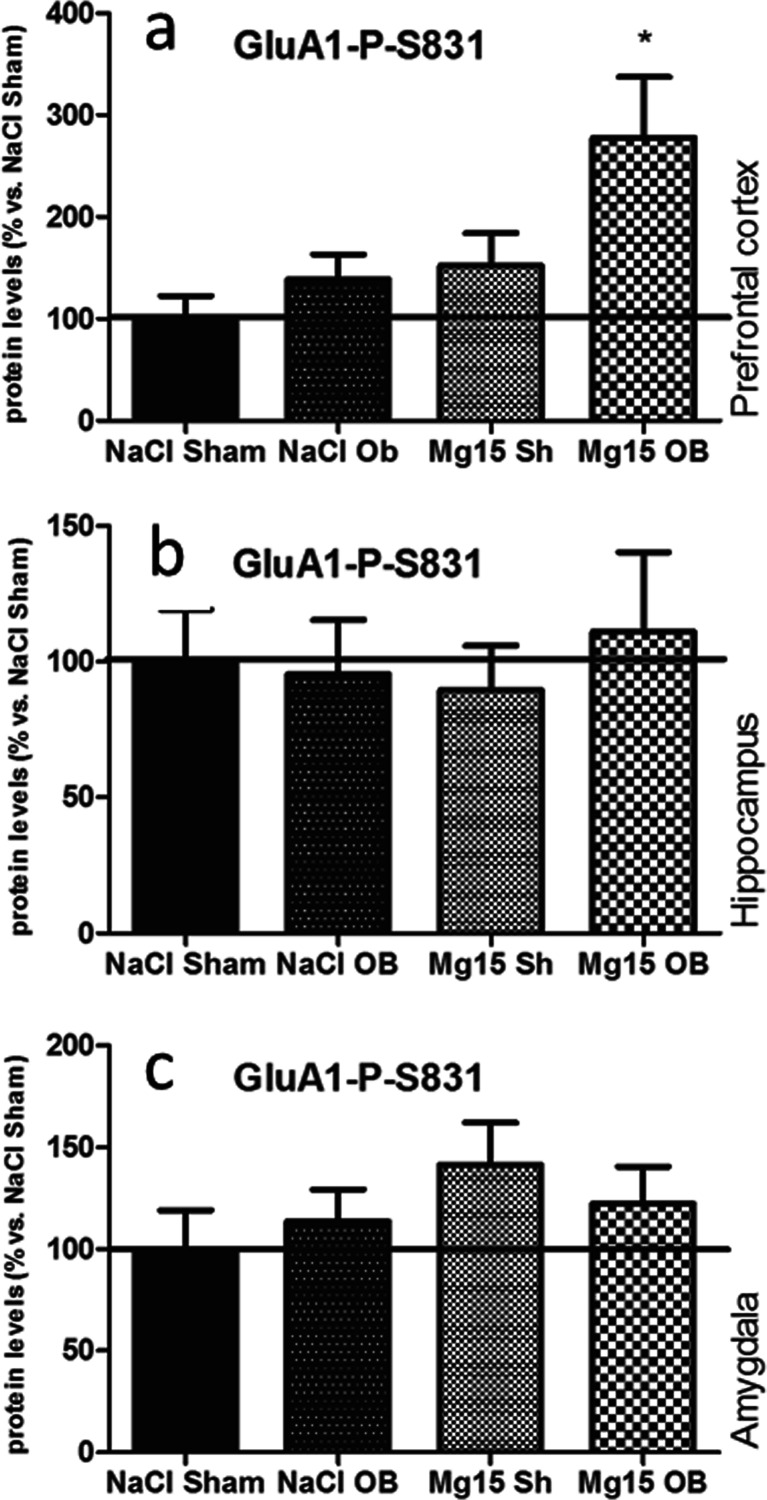
Changes in the protein levels of the phospho-Ser-831-GluA1 subunit of the AMPA receptor induced by OB and magnesium treatment. The levels of the phospho-Ser-831-GluA1 subunit were measured in the prefrontal cortex (**a**), the hippocampus (**b**), and amygdala (**c**) of sham and OB rats, treated for 2 weeks with a vehicle or magnesium. The values represent the mean ± SEM (*n* = 7–8). *p < 0.05 vs. OB

As shown in Fig. 6, the OB procedure and administration of magnesium did not induce significant effects in the P-S831 of GluA1 subunit levels in the PFC (a), the hippocampus (b), or the amygdala (c) with one exception, that is, when magnesium treatment in the OB animals induced an increase of P-S831 of the GluA1 subunit level in the PFC (177 % of NaCl sham, *p* < 0.05 vs. NaCl OB; Fig. 6a). In the PFC, two-way ANOVA indicated significant effects of OB [*F*(1, 22) = 5.15, *p* = 0.0335] and magnesium [*F*(1, 22) = 6.94, *p* = 0.0151], but no significant interaction [*F*(1, 22) = 1.43, *p* = 0.2446]. In the hippocampus, two-way ANOVA indicated that there were no significant effect of OB [*F*(1, 18 = 0.49, *p* = 0.4950] or magnesium [*F*(1, 18) = 0.16, *p* = 0.6934] and no significant interaction [*F*(1, 18) = 0.80, *p* = 0.3720]. In the amygdala, two-way ANOVA indicated that there were no significant effect of OB [*F*(1, 23 = 0.03, *p* = 0.8739] or magnesium [*F*(1, 23) = 1.83, *p* = 0.1892] and no significant interaction [*F*(1, 23) = 0.78, *p* = 0.3869].

### Effects of OB and administration of magnesium on the P-S845 of the GluA1 subunit level

**Fig. 7:**
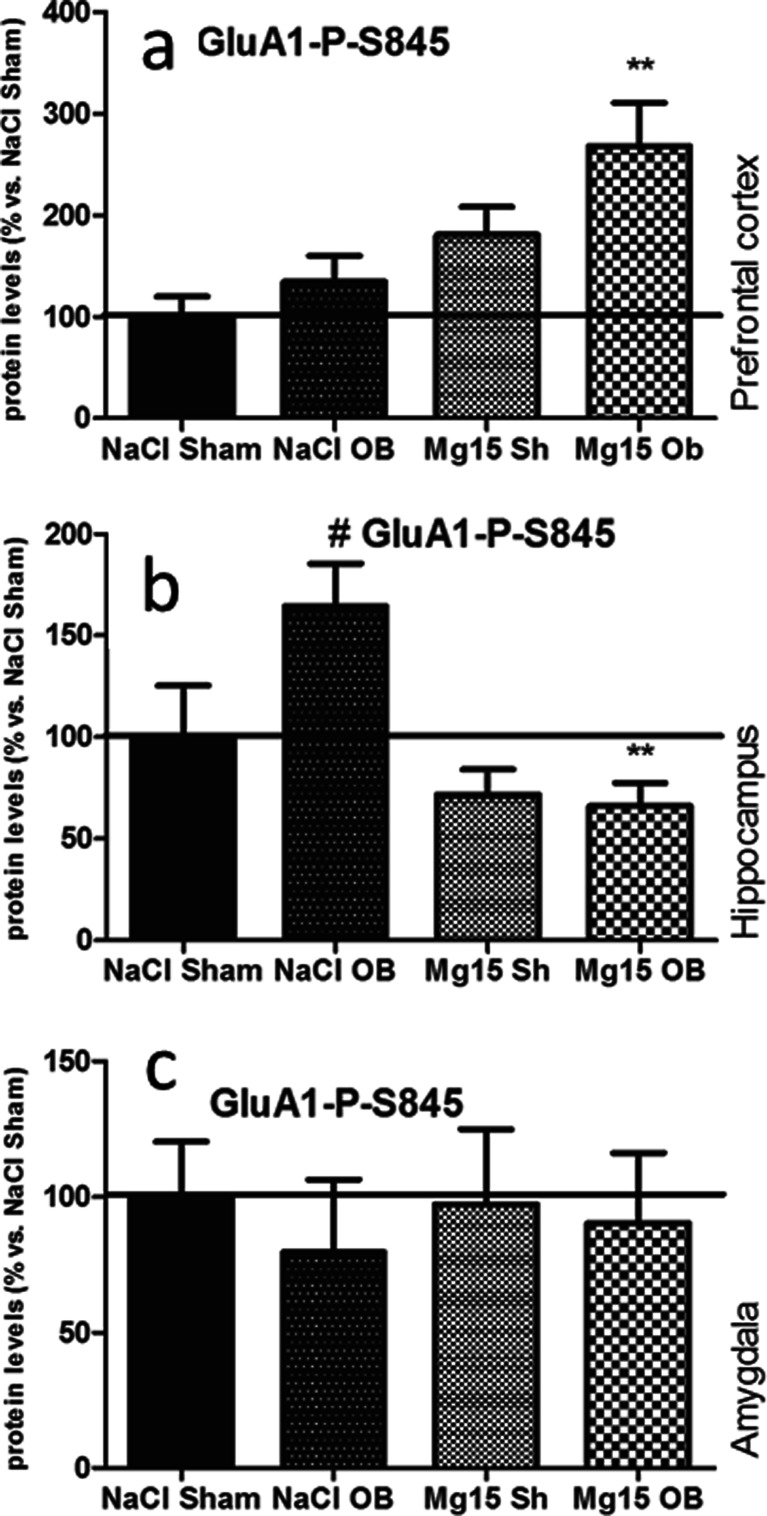
Changes in the protein levels of the phospho-Ser-845-GluA1 subunit of the AMPA receptor induced by OB and magnesium treatment. The levels of the phospho-Ser-845-GluA1 subunit were measured in the prefrontal cortex (**a**), the hippocampus (**b**), and amygdala (**c**) of sham and OB rats, treated for 2 weeks with a vehicle or magnesium. The values represent the mean ± SEM (*n* = 7–8). **p < 0.01 vs. OB, #*p* < 0.01 vs. sham

As shown in Fig. 7, the OB procedure and administration of magnesium did not induce significant effects in the P-S845 of GluA1 subunit levels in the PFC (a), the hippocampus (b), or the amygdala (c) with two exceptions, that is, when magnesium treatment in the OB animals induced increased P-S845 of the GluA1 subunit level in the PFC (168 % of NaCl sham, *p* < 0.01 vs. NaCl OB (a)) and when OB led to increased P-S845 in the hippocampus (164 % of NaCl sham, *p* < 0.05 vs. NaCl sham). This effect was reversed by magnesium (66 % of NaCl sham, *p* < 0.01 vs. NaCl OB (b)). In the PFC, two-way ANOVA indicated that there were no effect of OB [*F*(1, 24) = 4.01, *p* = 0.0567], a significant effect of magnesium [*F*(1, 24) = 12.53, *p* = 0.0017], and no significant interaction [*F*(1, 24) = 0.78, *p* = 0.3869]. In the hippocampus, two-way ANOVA indicated that there were no significant effect of OB [*F*(1, 22) = 2.78, *p* = 0.1090], a very significant effect of magnesium [*F*(1, 22) = 12.77, *p* = 0.0017], and a borderline interaction [*F*(1, 22) = 3.89, *p* = 0.0613]. In the amygdala, two-way ANOVA indicated that there were no significant effect of OB [*F*(1, 26 = 0.29, *p* = 0.5922], no significant effect of magnesium [*F*(1, 26) = 0.03, *p* = 0.8721], and no significant interaction [*F*(1, 26) = 0.07, *p* = 0.7932].

### Effects of OB and administration of magnesium on the BDNF level

**Fig. 8:**
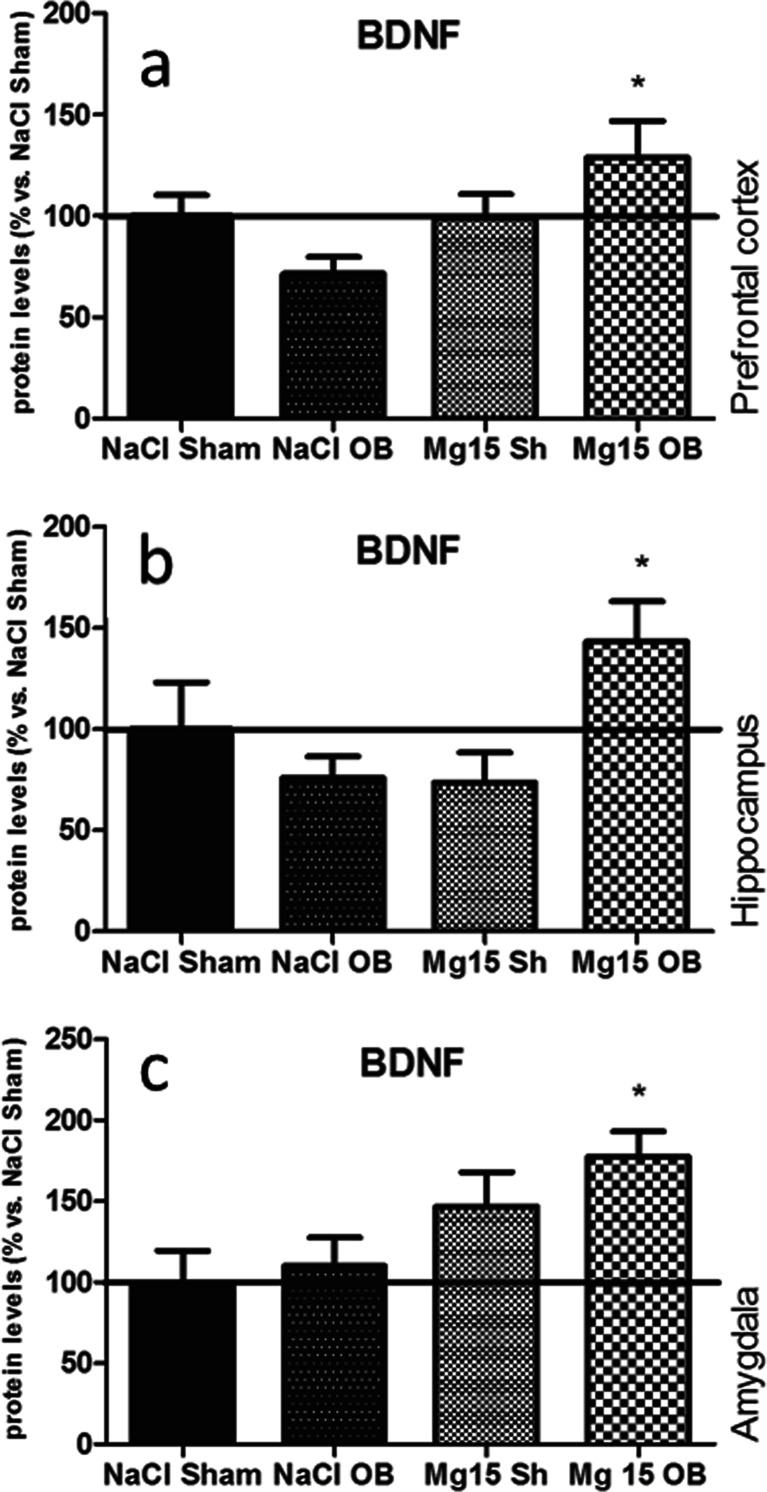
Changes in the protein levels of the BDNF induced by OB and magnesium treatment. The levels of the BDNF were measured in the prefrontal cortex (**a**), the hippocampus (**b**), and amygdala (**c**) of sham and OB rats, treated for 2 weeks with a vehicle or magnesium. The values represent the mean ± SEM (*n* = 7–8). *p < 0.05 vs. OB

As shown in Fig. 8, the OB procedure and administration of magnesium induced significant effects in the BDNF levels in the OB rats in all examined structures: the PFC (by 129 % of NaCl sham, *p* < 0.05 vs. NaCl OB) (a), the hippocampus (143 % of NaCl sham, *p* < 0.05 vs. NaCl OB) (b), and the amygdala (177 % of NaCl sham, *p* < 0.05 vs. NaCl OB) (c). In the PFC, two-way ANOVA indicated that there were no effect of OB [*F*(1, 22) = 0.01, *p* = 0.9433], a significant effect of magnesium [*F*(1, 22) = 4.57, *p* = 0.0438], and a significant interaction [*F*(1, 22) = 4.95, *p* = 0.0367]. In the hippocampus, two-way ANOVA indicated that there were no significant effect of the OB [*F*(1, 24) = 1.66, *p* = 0.2093], no significant effect of magnesium [*F*(1, 24) = 1.37, *p* = 0.2533], and a significant interaction [*F*(1, 24) = 7.13, *p* = 0.0134]. In the amygdala, two-way ANOVA indicated that there were no significant effect of OB [*F*(1, 24) = 1.19, *p* = 0.2870], a very significant effect of magnesium [*F*(1, 24) = 9.33, *p* = 0.0054], and no significant interaction [*F*(1, 24) = 0.3, *p* = 0.5870].

### Effects of OB and administration of magnesium on the GluN2A and GluN2B mRNA level

**Fig. 9:**
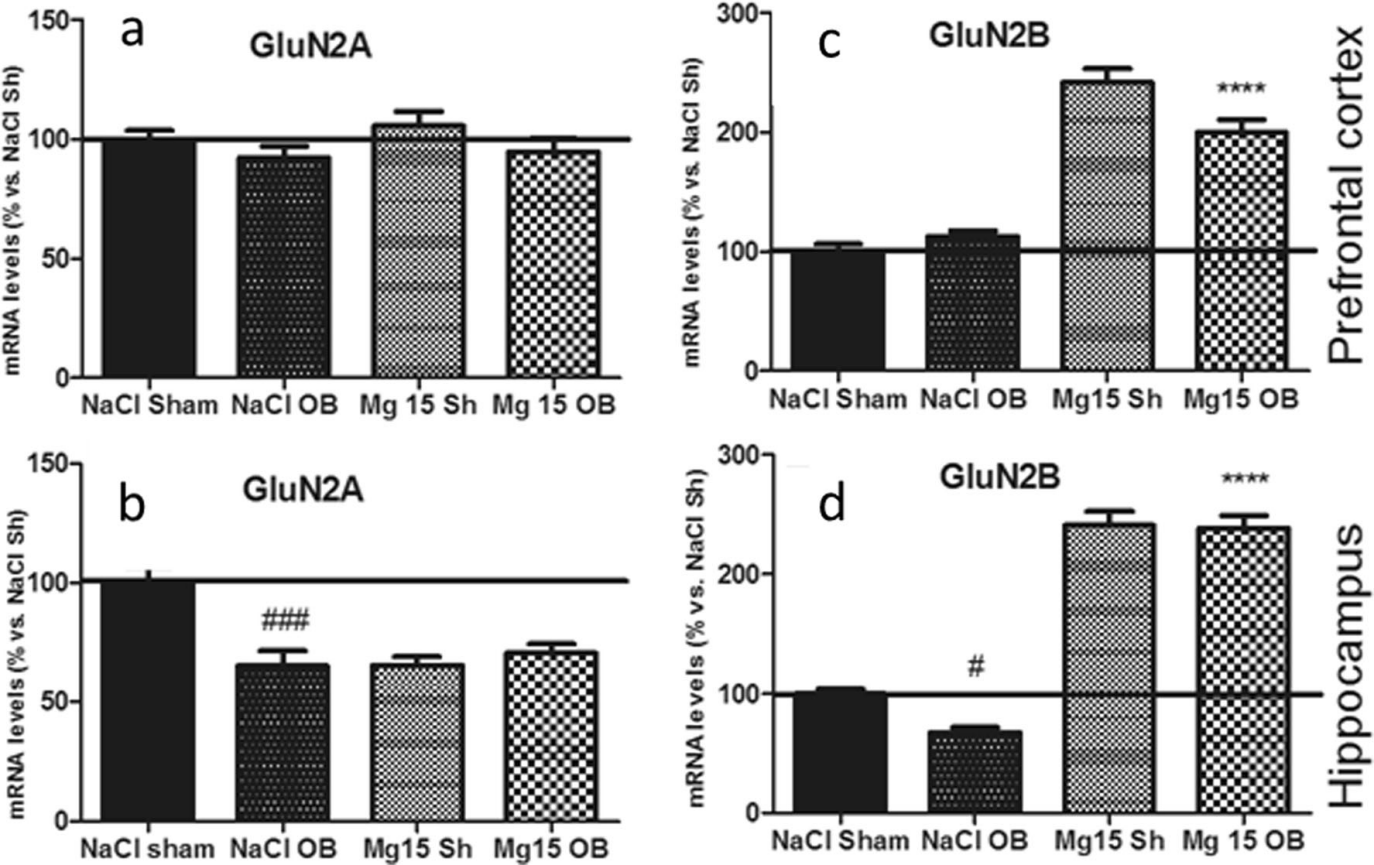
Changes in the mRNA levels of GluN2A and GluN2B subunits of NMDA receptors induced by OB and magnesium treatment. The levels of the mRNA level were measured in the PFC and hippocampus of sham and OB rats, treated for 2 weeks with a vehicle or magnesium. The values represent the mean ± SEM (*n* = 7–8). #*p* < 0.05 vs. sham, ###*p* < 0.001 vs. sham, ****p < 0.0001 vs. OB

As shown in Fig. 9, the OB procedure and administration of magnesium in all cases did not affect the level of the mRNA GluN2a subunit in the PFC. Two-way ANOVA indicated a borderline effect of OB [*F*(1, 22) = 3.12, *p* = 0.9144], no significant effect of magnesium [*F*(1, 22) = 0.55, *p* = 0.4654], and no significant interaction [*F*(1, 22) = 0.09, *p* = 0.7665]. As shown in Fig. 8b, the OB procedure significantly decreased the level of GluN2A mRNA in the hippocampus (*p* < 0.001 vs. NaCl sham). Magnesium did not reverse these changes. Two-way ANOVA indicated significant effects of OB [*F*(1, 26) = 7.05, *p* = 0.0134] and magnesium [*F*(1, 26) = 6.94, *p* = 0.0140] and a very significant interaction [*F*(1, 26) = 12.69, *p* = 0.0014]. As shown in Fig. 9c, the OB procedure did not affect the level of the GluN2B subunit mRNA level in the PFC, but the administration of magnesium in sham and OB animals led to a radical increase of the mRNA levels (*p* < 0.0001 vs. sham and *p* < 0.0001 vs. NaCl OB, respectively). Two-way ANOVA indicated that there were no significant effect of OB [*F*(1, 22) = 2.29, *p* = 0.1441], an extremely significant effect of magnesium [*F*(1, 22) = 142.84, *p* < 0.0001], and a very significant interaction [*F*(1, 22) = 8.06, *p* = 0.0095]. As shown in Fig. 9d, the OB procedure significantly decreased (*p* < 0.05 vs. NaCl sham) the GluN2B subunit mRNA level in the hippocampus, but the administration of magnesium in the sham and OB animals caused an extreme increase in the mRNA levels (*p* < 0.0001 vs. NaCl sham, *p* < 0.0001 vs. NaCl OB, respectively). Two-way ANOVA indicated a significant effect of OB [*F*(1, 26) = 4.74, *p* = 0.0387], an extremely significant effect of magnesium [*F*(1, 26) = 370.18, *p* < 0.0001], and a borderline interaction [*F*(1, 26) = 3.53, *p* = 0.0714].

## Discussion

In the present study, our findings provided new insights concerning the antidepressant-like activity of magnesium. We demonstrated that all tested doses of magnesium (10, 15, and 20 mg/kg) reduced hyperactivity in the open field test in rats in the olfactory bulbectomy (OB) model of depression. Because the increased hyperactivity in the open field test is considered a consequence of stress and/or anxiety (Harkin et al. 2003), these results appear to reinforce the general hypothesis that supports both the antidepressant and anxiolytic activities of magnesium (Poleszak et al. 2004; Poleszak et al. 2007).

In the present study, we also evaluated the magnesium effects in the passive avoidance test in the OB model. A deficit in the acquisition of the passive avoidance reflex is a well-established memory and learning disruption that results from OB (Kelly et al. 1997). In our study, the doses of 15 and 20 mg/kg of magnesium administered to OB rats were active and significantly reduced the number of trials required for the acquisition of a passive avoidance reflex compared with untreated OB rats. The efficacy of magnesium in the improvements of learning and memory observed in the passive avoidance test supports previous findings by Slutsky et al. (2010) who demonstrated the potential of magnesium to enhance the processes of memory and learning in rats (Slutsky et al. 2010). Lesion studies have conclusively demonstrated that the hippocampus and the amygdala are brain structures specifically involved in the acquisition of a passive avoidance reflex in rats (Slotnick 1973; Lorenzini et al. 1996; Ambrogi Lorenzini et al. 1997). Furthermore, the amygdala and the medial prefrontal cortex are also responsible for the formation of fear memory (LeDoux 2000).

Because OB impaired glutamate transmission in the amygdala, hippocampus, and cortical regions (Song and Leonard 2005; Van Riezen and Leonard 1990), we expected that the depressive-like symptoms induced by OB and/or the antidepressant-like activity of magnesium would be associated with the modulation of glutamatergic pathways. Our suppositions were enhanced by the fact that glutamate is a crucial neurotransmitter engaged in memory and learning processes, which are impaired by OB (Song and Leonard 2005). To verify this hypothesis, we studied the effects of OB and magnesium on the NMDAR and AMPAR receptor subunits. Unexpectedly, we did not identify differences in the levels of NMDAR and AMPAR subunits between the OB and sham animals in any studied brain structures. These findings may indicate the lack of an association between the levels of glutamate receptors and the depressive-like symptoms induced by OB. However, we cannot exclude that such changes may occur, although in a different region or level of the investigated brain structures.

On the other hand, the dose of 15 mg/kg of magnesium significantly elevated the GluN2B subunit and BDNF protein levels in the OB animals in the amygdala. These alterations may play an important role in the antidepressant-like activity of magnesium in OB, particularly in the passive avoidance task. There are no specific data that indicate the role of the glutamate system in the amygdala in the passive avoidance task, but many reports that concern conditioned fear emphasize the importance of NMDA-dependent transmission in the amygdala in fear learning (Miserendino et al. 1990; Goosens et al. 2003); for example, the injection of an NMDA antagonist into the amygdala inhibited the acquisition of fear conditioning (Miserendino et al. 1990). Furthermore, OB induces decreased binding of [^3^H] MK-801 to NMDARs in the amygdala (Ho et al. 2001). In addition, the studies of Slutsky et al. (2010) demonstrated that chronic administration of magnesium was associated with an increase in the BDNF and GluN2B subunit levels in the hippocampus, which indicates alterations that correlate with enhanced neuroplasticity. Therefore, we suggest that increased BDNF and GluN2B subunit levels in the amygdala may indicate the restoration of synaptic connections damaged by bulbectomy (Harkin et al. 2003; Song and Leonard 2005) and the normalization of neural transmission between the amygdala and other brain structures.

In contrast to the amygdala, the administration of magnesium did not lead to changes in the NMDAR subunit protein levels in the hippocampus or the PFC in OB rats. These results did not correlate with the mRNA levels. We determined that magnesium led to increased GluN2B subunit mRNA in the PFC and the hippocampus in the sham and OB rats. Furthermore, the mRNA levels of GluN2A and GluN2B subunits in the hippocampus of the OB rats were decreased compared with the sham animals. The lack of a similar pattern in the protein and mRNA expressions may be explained, in part, by the differences in the detection limits of the applied methods. Moreover, other factors, such as the translational regulation receptor half-life, can significantly contribute to the obtained results. Based on our previous studies, we also suspected that 2 weeks of magnesium administration was too short of a period to achieve elevated GluN2B subunits in the PFC. Previously, we demonstrated that 5 weeks of magnesium treatment in CMS animals led to an increased GluN2B protein level in the PFC (Pochwat et al. 2014).

We previously discussed that magnesium elevated the expression of BDNF in the amygdala in the OB animals. We also noticed similar events in the hippocampus and the PFC in the OB animals. However, it is difficult to interpret the enhanced expression of BDNF after magnesium treatment in the OB group as the reverse of OB-induced changes because we did not observe significant differences in the BDNF levels between the OB and sham animals. Our results are consistent with other studies performed in rats, which demonstrated no significant differences in the expression of BDNF at the protein (Luo et al. 2010) or mRNA levels (Van Hoomissen et al. 2003) after OB. Thus, the BDNF involvement in the depressive-like symptoms evoked by OB appears to be more complex.

Interestingly, we identified elevated levels of BDNF and the P-S831 and P-S845 of the GluA1 subunit of AMPARs in the PFC of the OB rats after magnesium treatment. The phosphorylation of these residues of serine represents crucial events for trafficking and inserting the GluA1 subunit of AMPARs into the synapse. AMPAR activation, together with BDNF release, is a fundamental condition for the processes of neuroplasticity (Lee et al. 2010; Lee and Kirkwood 2011; Duman and Voleti 2012). As has been shown in numerous studies, the insertion of the GluA1 subunit of AMPARs into the synapse, followed by the subsequent stimulation of the AMPAR GluA1 containing subunit, is a crucial event for the antidepressant mechanism of ketamine (Li et al. 2010), as well as drugs with a slow onset of action, such as fluoxetine (Svenningsson et al. 2002) or tianeptine (Svenningsson et al. 2007; Duman et al. 2012). These findings suggest that AMPAR activation, enhanced AMPAR transmission, and enhanced BDNF levels in the PFC may be potential mechanisms of action for the antidepressant-like activity of magnesium in OB rats. However, some issues require further studies.

**Fig. 10:**
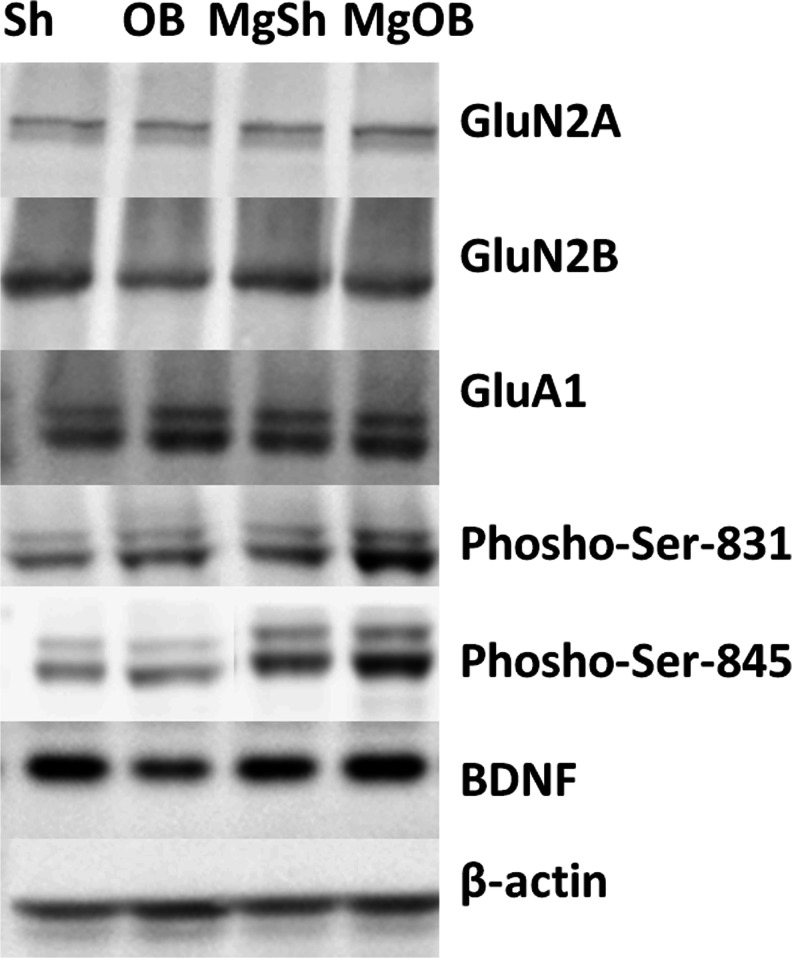
Representative Western blotting of NMDA receptor subunits (GluN2A, GluN2B), AMPA receptor subunits (GluA1, P-Ser-831, and P-Ser-845), BDNF, and β-actin in the PFC of a rat brain. The experimental conditions are described in “Material and methods”

It is well known that ketamine, which is also an NMDA antagonist, exerts its antidepressant-like activity in CMS after a single dose (Li et al. 2010). In our previous studies, we demonstrated that 3 weeks of treatment with magnesium was required to achieve the antidepressant-like activity in the CMS (Pochwat et al. 2014). On the other hand, it is well known that blockade of the NMDA receptor is essential for the antidepressant-like activity of a single dose of magnesium in the FST (Poleszak et al. 2007). It is likely that the molecular consequences of ketamine and magnesium administration, to some extent, may be similar, although the time required for their occurrence may be different. The administration of a very small dose of ketamine (0.5 mg/kg) required 11 days of treatment to achieve its antidepressant effect in the FST. This effect was associated with the phosphorylation of mTOR and an increased BDNF level in the hippocampus (Akinfiresoye and Tizabi 2013). It appears that despite the similar binding sites of ketamine and magnesium on NMDARs, different affinities of magnesium and ketamine to the NMDARs and differences in the pharmacokinetic profiles are the main factors that differentiate the onset of action and the profile of the antidepressant-like activity of these compounds (Orser et al. 1997).

In contrast to the PFC, we did not identify associations between increased BDNF levels and enhanced phosphorylation of AMPARs in the hippocampus. Additionally, we observed an increased level of P-S845 of the GluA1 in OB rats, which was attenuated by magnesium treatment. Several studies have revealed that P-S845 in the dentate gyrus of the hippocampus may be responsible for amphetamine-induced hyperactivity and the imipramine-dependent triggering of a manic state in bipolar disorder (Du et al. 2008). Magnesium’s ability to cause dephosphorylation of P-S845 of AMPARs may be a possible explanation of its potential to decrease hyperactivity in the open field test observed in OB rats. Using the data previously described as our basis, we suggest that the potential of magnesium to enhance the BDNF level in OB rats and the differentiated effects on AMPARs in the hippocampus and the PFC may be the primary mechanisms responsible for the effectiveness of magnesium in depression animal models, i.e., the OB model, as well as anxiety animal models.

To summarize, our present study demonstrated that magnesium reversed behavioral abnormalities, such as the deficit in the passive avoidance test or hyperactivity in the open field test, evoked by the removal of the olfactory bulbs. These effects were not accompanied by changes in the expression of GluN2A, GluN2B, or GluA1 subunits of glutamate ionotropic receptors in the examined brain structures. Although OB is an invasive procedure associated with neural damage, significant fluctuations in the BDNF levels have not been observed. Thus, direct involvement of these proteins in the induction of depressive-like behaviors appears to be irrelevant or at least unclear. The administration of magnesium in OB rats induced significant AMPAR alterations and increased BDNF levels in the PFC compared with the OB animals treated with saline. The BDNF level was also elevated in the hippocampus and the amygdala. However, AMPAR activation was only observed in the PFC, which indicates the functional relationship between these pathways. Combined with our previous results concerning magnesium activity in CMS, these findings suggest that magnesium is a potential therapeutic solution in patients with mood disorders associated with anhedonia, agitation, and anxiety.

## Abbreviations

AMPA: α-Amino-3-hydroxy-5 methyl-4-isoxazolepropionic acid
BDNF: Brain-derived neurotrophic factor
CaMKII: Ca2+/calmodulin-dependent protein kinase II
CMS: Chronic mild stress
CNS: Central nervous system
5,7-DCKA: 5,7-Dichlorokynurenic acid
FST: Forced swim test
MDD: Major depressive disorder
mTOR: Mammalian target of rapamycin
NMDA: *N*-methyl-d-aspartate
OB: Olfactory bulbectomy
PFC: Prefrontal cortex
PKA: Protein kinase A
PKC: Protein kinase C
P-S831: Phospho-Ser-831-GluA1
P-S845: Phospho-Ser-845-GluA1
RT-PCR: Real-time polymerase chain reaction
SDS: Sodium dodecyl sulfate
